# Effects of dust exposure on IGF1 and autophagy related to coal workers’ pneumoconiosis

**DOI:** 10.3389/fpubh.2026.1899729

**Published:** 2026-08-21

**Authors:** Yi Hu, Chunchao Zheng, Lei Chu, Xinyao Wang, Hailan He, Heliang Liu

**Affiliations:** 1 School of Public Health, North China University of Science and Technology, Tangshan, Hebei, China; 2 Hebei Key Laboratory of Organ Fibrosis, North China University of Science and Technology, Tangshan, Hebei, China

**Keywords:** autophagy, coal miners, correlated factors, insulin-like growth factor 1, pneumoconiosis

## Abstract

**Objective:**

To analyze the correlated factors of pneumoconiosis in coal miners, and explore the potential regulatory mechanism of insulin-like growth factor 1 (IGF1) in pneumoconiosis progression, so as to provide scientific evidence for the prevention and clinical treatment of coal workers’ pneumoconiosis.

**Methods:**

Clinical data of 2028 employees from a coal mining enterprise in Tangshan City, Hebei Province during 2023–2024 were retrospectively analyzed to screen the risk indicators of coal workers’ pneumoconiosis. A SiO₂-induced macrophage injury cell model was established to simulate pneumoconiosis-related pathological changes. The expression levels of inflammatory cytokines (IL-6, IL-1β), polypeptide growth factor (IGF1), autophagy marker proteins (LC3, P62) and intracellular signaling kinases (PI3K, AKT, mTOR) were detected. Logistic regression analysis was applied to identify pneumoconiosis correlated factors, and bioinformatics analysis was performed to clarify the regulatory relationship between dust exposure, IGF1 expression and cellular autophagy activity.

**Results:**

Early initial working age, long dust exposure duration, smoking, elevated white blood cell count and increased AST/ALT ratio were key correlated factors for pneumoconiosis. The coal worker pneumoconiosis group presented enhanced inflammatory response, upregulated IGF1 expression and altered autophagy-related protein expression activity. Consistent with the observational trends observed in human subjects, *in vitro* cellular assays demonstrated that SiO₂ stimulation was accompanied by elevated inflammatory response and IGF1 abundance, altered autophagy-related protein expression, and a rise in activity of the PI3K/AKT/mTOR kinase signaling cascade.

**Conclusion:**

Age, early initial working age, prolonged dust exposure, smoking, elevated white blood cell count and increased AST/ALT ratio are correlated factors for coal workers’ pneumoconiosis. Synchronous alterations in IGF1 upregulation, elevated phosphorylation levels of PI3K and AKT, and abnormal expression of autophagy-related markers were observed in pneumoconiosis patients and silica-stimulated macrophages, revealing correlative molecular links among these molecules in pneumoconiosis progression.

## 1 Introduction

Pneumoconiosis is a pulmonary disease caused by the inhalation of productive dust in occupational production and the retention of dust in the lungs, the pulmonary disease, characterized by persistent inflammation and diffuse fibrosis, is a very serious systemic occupational disease. Coal mines are typical high-incidence scenarios for pneumoconiosis, and workers exposed to coal mine dust face a significantly elevated risk of developing coal workers’ pneumoconiosis (CWP) (1). The incidence of CWP is high, and even if the dust exposure environment is removed, the pulmonary fibrosis will continue to develop and cannot be cured (2, 3). By the end of 2021, the number of reported cases of coal workers’ pneumoconiosis accounted for more than 80% of the total number of reported occupational diseases every year, this has seriously affected the health of our workers and the development of the national economy.

Existing studies have demonstrated that inhalable occupational dust (e.g., free silica particles) triggers a cascade of pulmonary stress responses once deposited in lung tissues. During dust phagocytosis, pulmonary macrophages suffer structural damage and eventual necrosis. The residual dust particles and necrotic cell debris are subsequently engulfed by recruited macrophages, forming an ongoing pathological vicious cycle. This process stimulates the persistent secretion of profibrotic cytokines, ultimately driving pulmonary fibrosis and silicotic nodule formation (4). Previous evidence has further verified that SiO₂ stimulation induces macrophage foaming, a key cellular phenotypic change closely implicated in the progression of pulmonary fibrosis (5). Progressive fibrotic lesions further elevate respiratory resistance and impair alveolar elasticity, resulting in deteriorated pulmonary ventilation function (6). As the primary immune effector cells in the lung, macrophages serve as the frontline defense against inhaled harmful particulate matter and are central to the maintenance of pulmonary homeostasis (7). This series of silica-induced macrophage injury and fibrotic pathological changes constitutes the core pathological basis of coal workers’ pneumoconiosis (CWP). Emerging studies indicate that insulin-like growth factor 1 (IGF1) modulates macrophage autophagy and inflammatory responses upon silica exposure, which may act as a vital molecular bridge linking dust stimulation to the progression of coal workers’ pneumoconiosis (7–9).

Growing research has identified autophagy as a critical regulatory mechanism governing CWP pathogenesis, with targeted modulation of macrophage autophagy proven to alleviate crystalline silica-induced mitochondrial apoptosis and abnormal inflammatory cytokine release in alveolar macrophages (7). Insulin-like growth factor 1 (IGF1), a 70-amino acid single-chain polypeptide with a molecular weight of 7.6 kDa, is endogenously synthesized and secreted by multiple human organs including the liver, kidney and spleen (8). Functionally, IGF1 exerts extensive biological regulatory effects, covering vasodilation, somatic growth promotion, cell proliferation and differentiation modulation, autophagy regulation, and tissue wound repair, and is also closely associated with tumor progression (8). Of particular relevance to CWP research, IGF1 acts as an upstream regulatory agonist that negatively modulates cellular autophagy activity (9). Given the indispensable role of macrophage autophagy in limiting pulmonary fibrotic injury and the regulatory function of IGF1 on autophagy and cell proliferation, exploring the interactive effects of IGF1 and autophagy on CWP progression is essential to clarify the molecular mechanism underlying CWP pathogenesis.

Despite the above preliminary findings, the combined regulatory mechanism of IGF1 and autophagy in CWP development remains poorly elucidated, with insufficient clinical and cellular evidence to support targeted intervention for CWP. To address this research gap, the present study integrated *in vitro* cellular experiments and clinical population analysis. A SiO_2_-stimulated THP-1 macrophage model was constructed to explore the cellular-level regulatory relationship between IGF1 and autophagy. After adjusting for confounding pneumoconiosis-related factors, this study systematically characterized the correlated expression changes of IGF1, autophagy and the PI3K/AKT/mTOR signaling pathway induced by silica exposure based on population epidemiological data and *in vitro* macrophage experiments. Combining bioinformatic transcriptional screening clues with population and cellular protein detection results, we identified synchronous molecular changes featuring elevated IGF1 expression, elevated phosphorylation levels of PI3K and AKT, and altered expression of autophagy-related proteins in silica-exposed coal miners and SiO₂-stimulated THP-1 macrophages. These correlative molecular changes provide preliminary observational clues for exploring potential molecular pathways underlying CWP progression.

## 2 Materials and methods

### 2.1 Main reagents and instruments

The silica was purchased from Sigma Corporation in the United States; RPM-1640 culture-medium purchased from Thermo Fisher Biochemical Products (Beijing) Limited company; Penicillin streptomycin double resistance was purchased from Gibco, USA; Protease inhibitors were purchased from Roche in Germany; BCA kit is purchased from Shanghai Biyuntian Limited company; Elisa (human IGF1, IL-6 and TNF-*α*) kits were purchased from ABclonal Inc.; The electrophoresis apparatus was purchased from BIO-RAD; The Human peripheral blood Monocytic Cells (Human Acute Monocytic Leukemia Cells, THP-1) cell line was purchased from the cell bank of Shanghai Institute of Biological Sciences, Chinese Academy of Sciences.

### 2.2 Study population

In this study, a cluster sampling method was used to select workers in a coal mine in Tangshan City, Hebei Province as the research object. Subjects with missing key variables including WBC, AST/ALT, occupational time indicators and smoking status were directly excluded during preliminary screening before formal screening based on unified inclusion and exclusion criteria, and multiple imputation or single value filling was not performed to handle missing observations in this study. Inclusion criteria: (1) Engaged in or ever engaged in coal mining, excavation, development, belt, coal washing and other production work; (2) Working experience of one year or more. Exclusion criteria: (1) Not cooperating with questionnaire investigators; (2) Refusal of physical examination; (3) People with other lung diseases (such as emphysema, asthma, etc.) (4) Patients with malignant tumors or other infectious diseases. This study was approved by the Ethics Committee of North China University of Science and Technology (approval number: 2021041), and carried out after signing the informed consent. Definition of relevant indicators: (1) Hypertension: systolic blood pressure ≥ 140 mm Hg or diastolic blood pressure ≥ 90 mm Hg, or a history of hypertension (10). (2) Body mass Index (BMI): BMI < 24.0 kg /m^2^ is defined as normal weight, 24.0 kg /m^2^ ≤ BMI < 28.0 kg /m^2^ is defined as overweight, BMI ≥ 28.0 kg/m^2^ is defined as obese (11). (3) Smoking: Smoking status was classified as never smoking in the past, smoking now and quitting (12). (4) Drinking: Drinking status was divided into never drinking in the past, drinking now or have stopped drinking (12). (5) Education level: junior high school education and below for junior, high school and technical school (technical school) for intermediate, college and above for senior (13). (6) Pneumoconiosis: According to the “Diagnosis of Occupational pneumoconiosis” (GBZ 70–2015) standard, the hospital diagnosed the patient as pneumoconiosis (14). (7) Definition of dust exposure: Total working years engaged in dust-generating underground posts (excavation, coal mining, coal washing, etc.) with continuous free silica exposure (14).

**Figure 1:**
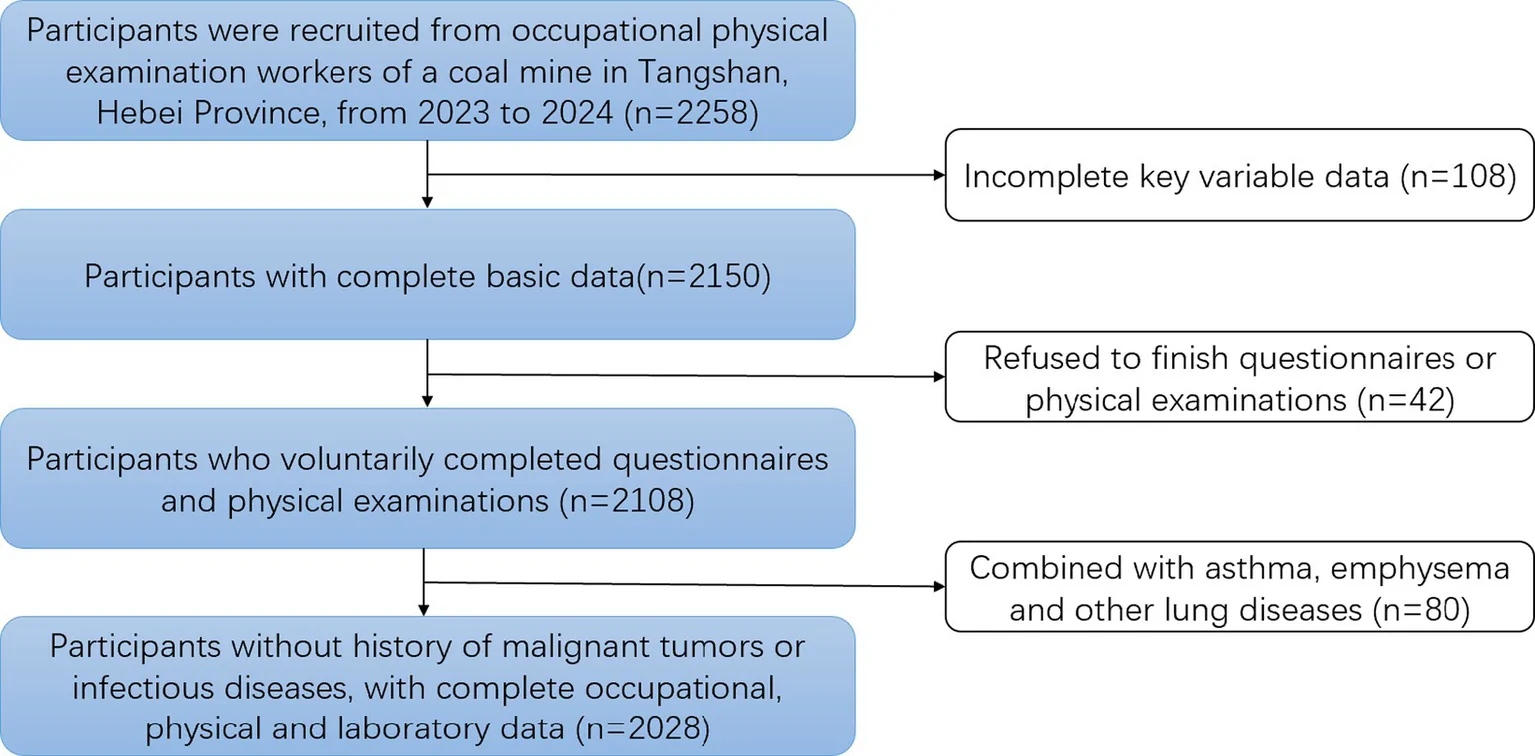
Flowchart of participant screening and inclusion.

A total of 2,258 coal mine workers with occupational physical examination data from 2023 to 2024 were set as initial research objects, and the screening process based on inclusion and exclusion criteria is shown in Figure 1. After screening according to inclusion and exclusion criteria, 2028 research objects were finally included for subsequent analysis. All research objects were divided into four subgroups according to dust exposure history and the GBZ 70–2015 pneumoconiosis diagnostic standard (14): non-dust-exposed control group, ongoing silica dust exposure group, dust-withdrawn group, and coal workers’ pneumoconiosis case group. Fifty-five research objects covering all four subgroups were randomly selected from the 2028 participants to measure plasma IGF1, IL-6 and TNF-*α* via ELISA. These 55 subjects also provided PBMC specimens for subsequent Western blotting assays, with detailed subgroup allocation as follows based on dust exposure history and pneumoconiosis diagnosis: 9 dust-free healthy miners in the control group, 26 workers with long-term ongoing dust exposure but no pneumoconiosis in the dust-exposed group, 8 individuals transferred away from dust posts for over one year in the dust-removed group, and 12 clinically confirmed pneumoconiosis patients in the case group.

### 2.3 Research method

#### 2.3.1 Baseline data collection

(1) Questionnaire survey: The baseline data of coal miners were collected by questionnaire survey, including general demographic information, occupational history, disease history, psychological state, etc. (2) Physical examination: Blood and urine samples of the subjects were collected, and biochemical data, blood pressure, height, weight and other relevant indicators of the workers were detected and recorded. (3) Laboratory detection: Plasma levels of inflammatory factors IL-6 and TNF-*α* were detected by enzyme-linked immunosorbent assay (ELisa) kit.

#### 2.3.2 Plasma sample collection

During physical examination, venous blood was collected using 10 mL sampling vessels from all 2028 participants. After centrifugation, the upper plasma was collected and divided into four 1.5 mL centrifugal tubes, coded according to the sequence, and stored in the refrigerator at −80 °C. Among all reserved plasma specimens, 55 samples covering four exposure subgroups were randomly selected for subsequent ELISA detection of IGF1, IL-6 and TNF-*α*.

#### 2.3.3 Isolation of human peripheral blood mononuclear cells (PBMCs) and total protein extraction

Peripheral blood mononuclear cells (PBMCs) were adopted as human biological samples for Western blotting analysis in this study. Fasting venous blood samples were collected from male coal miners including control workers, dust-exposed workers, workers who had stopped dust exposure, and diagnosed pneumoconiosis patients. The collected whole blood was centrifuged at 400 G for 10 min to separate plasma. The remaining blood cell suspension was slowly overlaid onto human peripheral blood lymphocyte separation medium, followed by centrifugation at 600 G for 30 min. The cloudy intermediate layer containing PBMCs was carefully aspirated, and the harvested PBMC precipitate was washed three times with calcium- and magnesium-free PBS after centrifugation at 500 G for 10 min each time. After washing, PBMC pellets were lysed with RIPA lysis buffer supplemented with protease inhibitor cocktail on ice, then subjected to sonication for 10 s and incubated on ice for 30 min to extract total cellular protein. The lysate was centrifuged at 12,000 rpm at 4 °C for 20 min, and the supernatant containing total protein was aliquoted and stored at −80 °C for subsequent Western blotting detection. Each lane on the Western blot gel was loaded with protein lysate obtained from PBMCs of one separate individual participant, and no pooled mixed samples were used.

### 2.4 Bioinformatics analysis

In this study, Three GEO datasets (GSE29110, GSE188520, GSE245655) were included. GSE29110 contains 6 rat lung tissue samples (3 SiO₂-treated, 3 controls) generated on GPL1355. GSE188520 contains 6 rat lung tissue samples (3 SiO₂-treated, 3 controls) generated on GPL25290. GSE245655 contains 9 mouse lung tissue samples (3 controls, 3 SiO₂-treated, and 3 SiO₂ + ATL-III) generated on GPL13112. We extracted IGF1 expression values from each dataset and standardized raw data uniformly. log_2_(x + 1) transformation was applied to eliminate technical differences across samples and improve comparability between datasets.

The three datasets were selected for their complementary strengths and high compatibility with coal workers’ pneumoconiosis (CWP) and fibrosis research. GSE29110 characterizes transcriptomic changes induced by long-term coal dust exposure; GSE188520 reflects gene alterations in silica-induced pulmonary fibrosis; GSE245655 profiles transcriptomic features of progressive pneumoconiosis. Combined analysis covers dust-induced injury, fibrotic lesion formation and disease progression, reducing single-dataset bias and improving screening robustness.

The standardized analytical workflow was performed as follows. Raw expression matrices of the three datasets were downloaded, normalized, and batch-effect corrected in R 4.2.3. DEGs were screened between pneumoconiosis and control groups with thresholds of |logFC| > 1 and *p* < 0.05. Overlapping DEGs across the three datasets were identified via the “VennDiagram” package as core candidate genes. GO and KEGG enrichment analyses were further performed to annotate the biological functions and signaling pathways of core DEGs, focusing on inflammation, autophagy and fibrosis-related pathways.

### 2.5 Cell modeling

THP-1 cells in good growth condition and logarithmic growth phase were selected for subsequent experiments. Two experimental groups were established in this study: (1) Control group: THP-1 cells were cultured conventionally without additional intervention; (2) SiO₂-stimulated model group: THP-1 cells were seeded at a density of 1 × 10^5^ cells, followed by 48 h continuous treatment with 50 ng/mL PMA to induce adherent differentiation. After differentiation, the culture medium was refreshed, and differentiated macrophages were treated with 50 μg/mL SiO₂ suspension. Total cellular protein was harvested 48 h after SiO₂ stimulation for subsequent detection.

### 2.6 Western blot

Cells were rinsed with phosphate-buffered saline (PBS) and lysed on ice for 30 min with cell lysis buffer to extract total cellular protein. Subsequently, cell lysates were centrifuged at 12000 rpm at 4 °C for 10 min, and the supernatants were collected. The total protein concentration was quantified using a BCA protein assay kit (Thermo Fisher, 23,227). After adding 5 × protein loading buffer, the protein samples were heat-denatured, separated by sodium dodecyl sulfate-polyacrylamide gel electrophoresis (SDS-PAGE), and electrotransferred onto a nitrocellulose (NC) membrane. The membrane was blocked with 5% skim milk prepared in Tris-buffered saline containing 0.05% Tween-20 (TBST) for 2 h at room temperature. After blocking, the membrane was incubated with primary antibodies overnight at 4 °C with the following dilution ratios: *β*-Actin (1,3,000), IGF1 (1:1500), AKT (1:1500), p-AKT (1:1500), PI3K (1:1500), IL-6 (1:1500), TNF-*α* (1:1500), mTOR (1:1500), P62 (1:1500), and LC3 (1,1,500). On the next day, the membrane was washed thoroughly with TBST and incubated with corresponding secondary antibodies (rabbit antibody, 1:3000; mouse antibody, 1:3000) at room temperature for 2 h. Protein bands were visualized and captured using a fully automated ECL chemiluminescence imaging system (ChemiScope, Shanghai Qinxiang Scientific Instrument Co., Ltd., Shanghai, China). Finally, the gray values of all protein bands were quantitatively analyzed using ImageJ software.

### 2.7 Statistical analysis

All questionnaire data were double-entered and verified in the Epidata database to avoid input errors. Normally distributed continuous data were expressed as mean ± standard deviation and analyzed using independent t-test and analysis of variance, while non-normal data were presented as median (interquartile range) and analyzed by Kruskal–Wallis test. All datasets were tested for normality, independence and variance homogeneity before comparison. Count data were described as proportions and compared using the Chi-square test or Fisher’s exact test.

Binary logistic regression models were adopted to identify factors associated with coal workers’ pneumoconiosis; pneumoconiosis diagnosis was set as the binary dependent variable, while demographic, occupational and clinical indicators served as independent variables.

First, all variables with *p* < 0.2 in univariate analyses, along with age, age at first employment, dust exposure duration and working seniority, were preliminarily enrolled into candidate multivariate models. VIF tests were subsequently performed to assess multicollinearity, and no severe multicollinearity was observed (all VIF values < 10), despite moderate correlations among occupational exposure duration-related indicators. To eliminate confounding bias caused by age, this indicator was retained in the final multivariate model.

After variable screening and collinearity verification, multivariate logistic regression was conducted to estimate correlations via odds ratios (OR) and corresponding 95% confidence intervals (95%CI). This model further adjusted for confounding factors including smoking status, working seniority and AST/ALT ratio when exploring the relationships between dust exposure, IGF1 levels and coal workers’ pneumoconiosis. All statistical analyses were carried out using SPSS 23.0 software, and a two-tailed *p* < 0.05 was defined as statistically significant.

For missing data, participants with missing key variables (WBC, AST/ALT, occupational indicators and smoking status) were excluded before modeling, and no data imputation was performed. Model calibration and fit were assessed via the Hosmer–Lemeshow test (*p* > 0.05) and low AIC/BIC values, indicating acceptable model performance.

## 3 Results

### 3.1 Basic information of the research object

The study subjects were all male, with an average age of 43.10 ± 8.55 years, an initial working age of 25.99 ± 6.34 years, an average dust exposure life of 14.50 ± 9.29 years, an average BMI of 26.11 ± 3.72 kg/m2, and an average working age of 16.99 ± 8.48 years. Among them, 1,144 workers smoked and 1,142 workers drank alcohol, accounting for 56.41 and 56.31%, respectively. The number of workers aged 30 to 40 was 680, accounting for 33.53%; The number of workers between 10 years and 20 years is 841, accounting for 41.47%; The number of workers suffering from hypertension and fatty liver was 786 and 1,062, accounting for 38.76 and 52.37%, respectively. The study found that the number of workers with BMI at the overweight level was 985, accounting for 48.57%. The subjects were divided into pneumoconiosis group and non-pneumoconiosis group according to whether they had pneumoconiosis or not. There were differences in age, initial working age, BMI, smoking, white blood cell count (WBC), aspartate aminotransferase (AST), total cholesterol (TC), AST/ALT alcohol consumption and hypertension between the two groups. The mean age, length of service, dust exposure period and white blood cell count (WBC) of the pneumoconiosis group were higher than those of the non-pneumoconiosis group (*p* < 0.05) (Tables 1, 2).

**Table 1:** Association between pneumoconiosis and physical examination index.

Variable	$\bar{x}$	Nonpneumoconiosis	Pneumoconiosis	*t*	*P*
		$\bar{x}$	$\bar{x}$		
Age	43.10	42.19 ± 0.178	56.00 ± 0.803	−19.636	<0.0001
Initial working age	25.99	25.73 ± 0.138	29.67 ± 0.798	−4.86	<0.0001
Dust exposure period	14.50	13.99 ± 0.208	21.78 ± 0.818	−9.551	<0.0001
BMI	26.11	26.21 ± 0.849	24.73 ± 0.323	4.453	<0.0001
Length of service	16.99	16.65 ± 0.191	21.82 ± 0.815	−6.181	<0.0001
WBC	6.36	6.34 ± 0.038	6.70 ± 0.159	−2.103	0.036
AST	24.14	24.38 ± 0.228	20.74 ± 0.625	4.148	<0.0001
TC	4.78	4.79 ± 0.233	4.56 ± 0.072	2.539	0.011
AST/ALT	1.05	1.06 ± 0.009	0.94 ± 0.355	3.109	0.002

**Table 2:** Analysis of pneumoconiosis and population characteristics.

Project	Group	Constituent ratio (%)	Nonpneumoconiosis	Pneumoconiosis	χ^2^	*P*
			*N* (%)	*N* (%)		
Smoking	Non-smoking	770 (37.97)	743 (96.49)	27 (3.51)	25.3	<0.001
	Smoking	1,144 (56.41)	1,054 (92.13)	90 (7.87)		
	ex-smokers	114 (5.62)	98 (85.96)	16 (14.04)		
Drink	Non-drinking	833 (41.07)	781 (93.76)	52 (6.24)	6.49	0.039
	Drinking	1,142 (56.31)	1,069 (93.61)	73 (6.39)		
	Temperance	53 (2.61)	45 (84.91)	8 (15.09)		
Age	~30	613 (30.23)	613 (100)	0 (0)	495.83	<0.001
	30 ~ 40	680 (33.53)	677 (99.56)	3 (0.44)		
	40 ~ 50	475 (23.42)	443 (93.26)	32 (6.74)		
	50~	260 (12.83)	162 (62.31)	98 (37.69)		
Hypertension	No	1,242 (61.24)	1,147 (92.35)	95 (7.65)	6.22	0.013
	Yes	786 (38.76)	748 (95.17)	38 (4.83)		
Length of service	~10	412 (30.32)	398 (96.6)	14 (3.4)	79.7	<0.001
	10 ~ 20	975 (48.08)	940 (96.41)	35 (3.59)		
	20 ~ 30	435 (21.45)	389 (89.43)	46 (10.57)		
	30~	206 (10.16)	168 (81.55)	38 (18.45)		
BMI	Normal	605 (29.83)	546 (90.25)	59 (9.75)	14.52	0.001
	Overweight	985 (48.57)	932 (94.62)	53 (5.38)		
	Obesity	438 (21.60)	417 (95.21)	21 (4.79)		
Fatty liver	No	966 (47.63)	893 (92.44)	73 (7.56)	3.003	0.083
	Yes	1,062 (52.37)	1,002 (94.35)	60 (5.65)		

### 3.2 Analysis of correlated factors for pneumoconiosis

The logistic regression results clearly demonstrated that age, initial working age, dust exposure duration, smoking status, elevated WBC count, abnormal AST/ALT ratio, and prolonged working seniority were all relevant factors for CWP. Quantitative interpretation of each index was as follows: each additional year of age increased the prevalence risk of pneumoconiosis by 19.3%. Each additional year of dust exposure duration increased the prevalence risk of pneumoconiosis by 6.7%. In addition, initial working age was also positively associated with CWP risk, with each additional year of age at first dust exposure corresponding to an 11.1% increase in prevalence odds. This finding suggests that older age at first entry into dust-exposed posts may confer greater susceptibility to dust-induced lung injury. In terms of smoking-related risk, current smokers and former smokers exhibited 2.604-fold and 2.381-fold higher risks of developing pneumoconiosis compared with never-smokers. For laboratory indicators, every one-unit elevation in peripheral WBC count increased the risk of CWP by 18.5%, and participants with an abnormal AST/ALT ratio had a 3.524-fold higher pneumoconiosis risk than those with normal liver enzyme levels (Tables 3, 4).

**Table 3:** Logistic regression analysis model variable assignment.

Factor	Variable	Assign
Pneumoconiosis or not	Y	No = 0, Yes = 1
Age	X1	
Initial working age	X2	
Dust exposure period	X3	
Smoking	X4	No smoking = 0, Smoking = 1, Smoking cessation = 2
WBC	X5	
AST/ALT	X6	Normal = 0, Abnormal = 1
length of service	X7	~10=1, 10 ~ 20 = 2, 20 ~ 30 = 3, 30 ~ =4

**Table 4:** Analysis of correlated factors for pneumoconiosis.

Factor	Group	Reference	Beta	SE	χ^2^	*P*	OR (95%CI)
Age			0.176	0.021	67.601	<0.001	1.193 (1.144–1.244)
Initial working age			0.105	0.026	15.926	<0.001	1.111 (1.055–1.170)
Dust exposure period			0.065	0.019	11.879	0.001	1.067 (1.028–1.107)
Smoking	Smoking	No smoking	0.957	0.261	13.451	<0.001	2.604 (1.561–4.342)
	Ex-smokers		0.867	0.419	4.276	0.039	2.381 (1.046–5.416)
WBC			0.169	0.066	6.583	0.010	1.185 (1.041–1.348)
AST/ALT	Normal	Abnormal	1.260	0.228	30.585	<0.001	3.524 (2.255–5.507)
Length of service	10 ~ 20	~10	−0.191	0.453	5.790	0.016	0.336 (0.138–0.817)
	20 ~ 30		−0.133	0.581	0.052	0.819	0.875 (0.280–2.736)
	30~		−0.688	0.812	0.717	0.397	0.503 (0.102–2.471)

Furthermore, the association between working seniority and CWP prevalence was inconsistent across subgroups when adjusted for other confounding factors. Taking participants with less than 10 years of service as the reference group, only workers with 10–20 years of service exhibited a statistically significant difference, which showed a lower odds of pneumoconiosis (OR = 0.336, 95%CI: 0.138–0.817, *p* = 0.016), suggesting this stratum acted as a protective factor in this model. No statistically significant correlations with pneumoconiosis risk were detected for participants with 20–30 years or more than 30 years of working seniority (both *p* > 0.05, Table 4).

To eliminate the interference of independent variable multicollinearity on the stability of multivariate Logistic regression model, collinearity diagnosis was conducted (Table 5). The results showed that the variance inflation factor (VIF) of all included variables was less than 10. The VIF value of age was 3.808, initial working age was 3.031, dust exposure period was 2.037, and the VIF values of smoking, WBC, AST/ALT and working seniority were close to 1. All Tolerance values were higher than 0.2. The overall model presented *F* = 69.237, *R^2^* = 0.194, *P* < 0.001.

**Table 5:** Collinearity diagnostics for study-related variables.

Variable	*β*	SE	*β*-standardized	*t*	*P*	95% CI Lower	95% CI Upper	Tolerance	VIF
Constant	−0.553	0.039	-	−13.738	<0.001	−0.609	−0.457	-	-
Age	0.013	0.001	0.438	11.229	<0.001	0.010	0.015	0.263	3.808
Initial working age	7.885E-6	0.001	0.000	0.006	0.995	−0.003	−0.003	0.330	3.031
Dust exposure period	0.002	0.001	0.059	2.066	0.039	0.000	0.003	0.491	2.037
Smoking	0.041	0.009	0.096	4.771	<0.001	0.024	0.058	0.990	1.010
WBC	0.008	0.003	0.053	2.617	0.009	0.002	0.014	0.974	1.027
AST/ALT	0.063	0.011	0.118	5.864	<0.001	0.042	0.085	0.984	1.017
Length of service	−0.031	0.011	−0.110	−2.701	0.007	−0.053	−0.008	0.974	1.027

VIF, variance inflation factor. The overall model: *F* = 69.237, *R*^2^ = 0.194, *p* < 0.001.

### 3.3 Bioinformatics analysis

**Figure 2:**
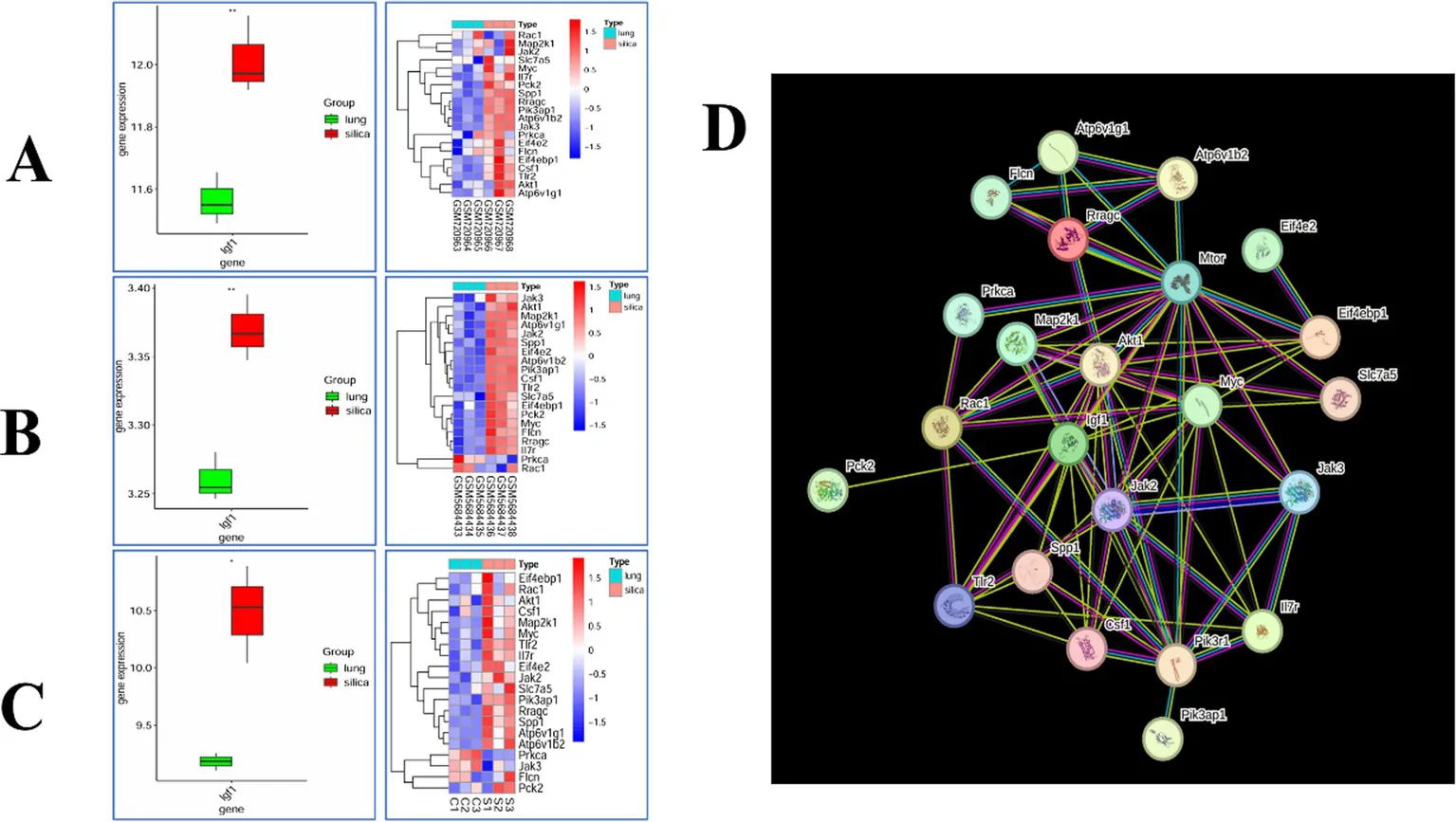
**(A)** GSE29110 data set; **(B)** GSE188520 data set; **(C)** GSE245655 data set; **(D)** protein interaction.

Systematic bioinformatics analysis based on three GEO pneumoconiosis transcriptome datasets was conducted to screen core genes relevant to CWP. Overlapping upregulated DEGs across the three datasets were imported into STRING to construct a PPI network. Six gene clusters containing 60 core genes were extracted via Cytoscape MCODE plugin, and IGF1 was identified as a key hub gene by CytoHubba (Figure 2).

GO and KEGG enrichment combined with PPI network analysis suggested significant mRNA upregulation of IGF1, PI3K, AKT and mTOR at the transcriptional level in silica-induced pneumoconiosis samples, and protein interaction prediction predicted direct molecular connections among the four molecules, hinting this signaling axis may participate in pneumoconiosis pathogenesis. Boxplots and heatmaps in Figures 2A–C showed elevated expression of these genes in silica-damaged lung tissues relative to normal controls. Figure 2D displayed the top-scoring gene cluster PPI network, where IGF1, PI3K, AKT and mTOR served as central nodes connected to fibrosis- and autophagy-related genes.

Transcriptomic data integrated from multiple datasets suggested elevated mRNA expression of IGF1, PI3K, AKT and mTOR in pneumoconiosis lesions, which implied potential involvement of the PI3K/AKT/mTOR cascade in disease-related pathological processes.

### 3.4 IGF1 levels and levels of inflammatory cytokines IL-6 and TNF-*α* were increased in the case group

**Figure 3:**
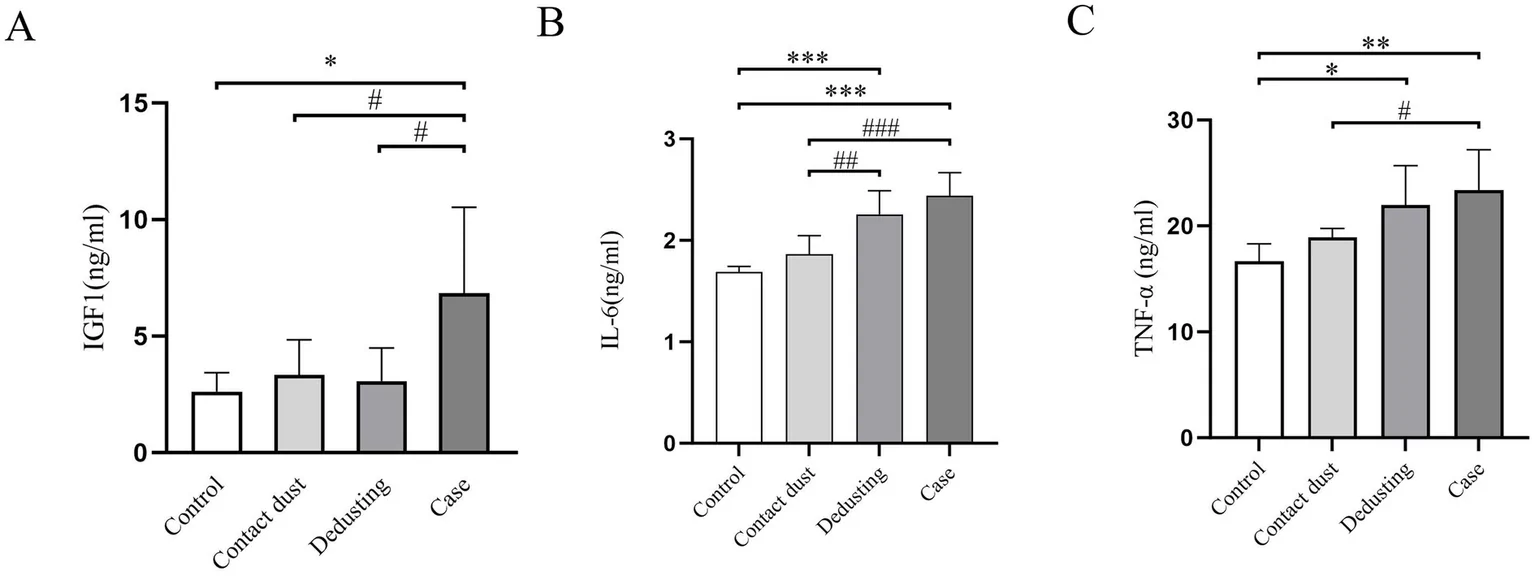
Detection of IGF1, IL-6 and TNF-*α* levels in human plasma. **(A)** ELISA results of IGF1 level in plasma, **(B)** ELISA results of IL-6 level in plasma, **(C)** ELISA results of TNF-α level in plasma. ^*^p < 0.05; ^**^p < 0.01; ^***^p < 0.001 versus the control group; *^#^p* < 0.05; *^##^p* < 0.01; *^###^p* < 0.001 versus the Case group.

Excluding the influence of smoking, the ratio of glutamate-grass to glutamate-C and working age, the human samples were divided into control group, dust exposure group, dust removal group and pneumoconiosis group according to dust exposure and whether or not pneumoconiosis was detected. ELisa kit was used to detect the levels of IGF1, IL-6 and TNF-*α* in human plasma samples. It was found that the levels of inflammatory cytokines IL-6 and TNF-α were gradually increased in control group, dust exposure group, dust removal group and case group. Compared with the control group, there were significant differences in IL-6 and TNF-α levels between the dust-removal group and the case group (*p* < 0.05). IGF1 levels also increased in the four groups. Compared with case group, there were differences in control group, dust exposure group and dust removal group, and the differences were statistically significant (*p* < 0.05) (Figure 3).

### 3.5 The autophagy level was significantly decreased in the case group

**Figure 4:**
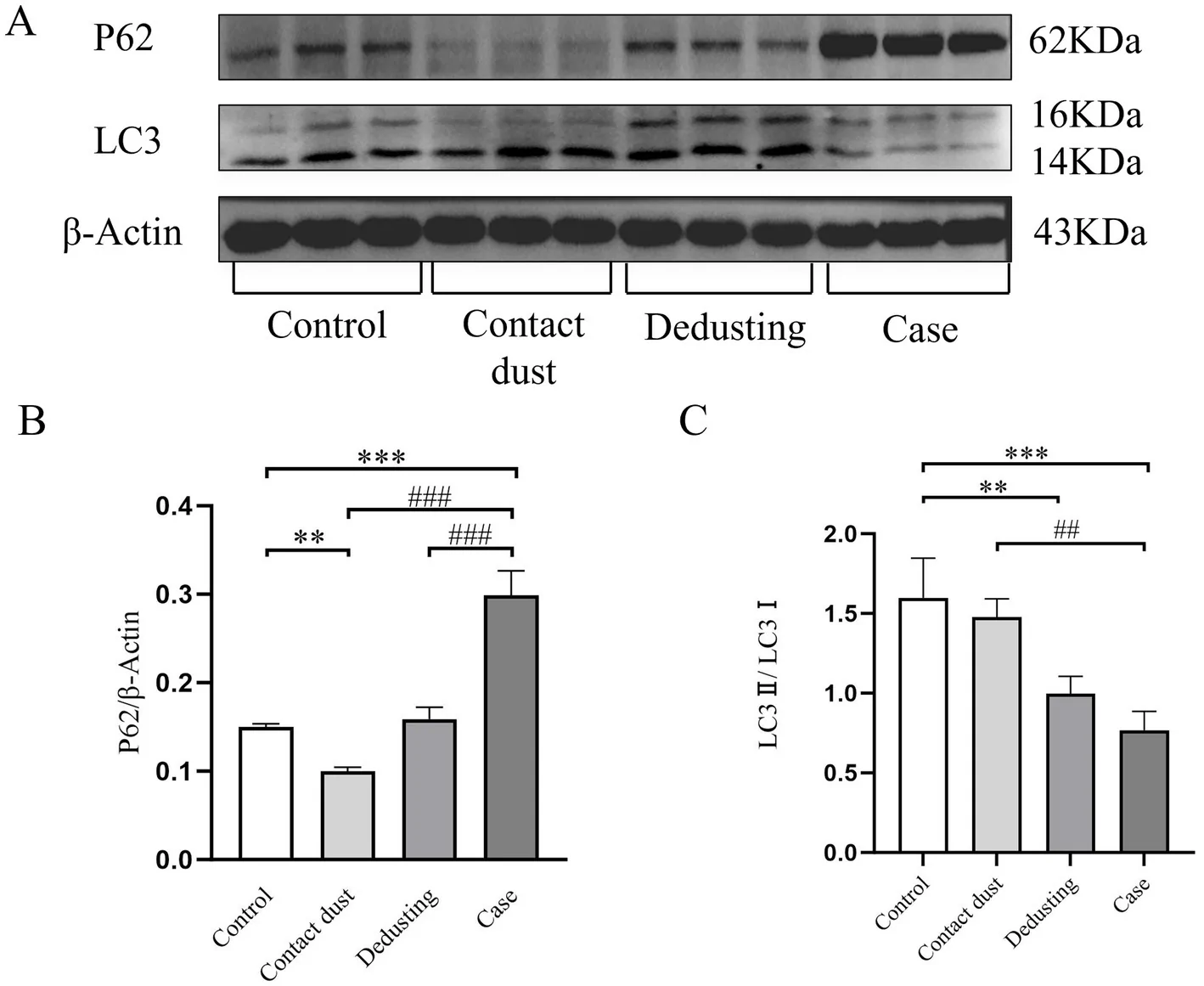
Human autophagy level detection. **(A–C)** Western blot for P62 and LC3 expression in the human sample. β-Actin was used as loading control. Datas are expressed as mean ± SD from at least three independent experiments in triplicate. ^*^p < 0.05; ^**^p < 0.01; ^***^p < 0.001 versus the control group; *^#^p* < 0.05; *^##^p* < 0.01; *^###^p* < 0.001 versus the ASEase group.

Western blot assay was used to detect autophagy marker protein P62 and LC3. In the control group, dust exposure group, dust removal group and case group, the accumulation of autophagy substrate P62 in the case group was significantly higher than that in the other three groups. At the same time, the values of autophagy markers LC3II/LC3I in control group, dust exposure group, dust removal group and case group were in a decreasing trend, and there were significant differences in autophagy levels between dust removal group and case group compared with control group, and there were also significant differences in autophagy levels between case group and dust exposure group, with statistical significance (*p* < 0.05) (Figure 4).

### 3.6 In the model group, the levels of inflammatory factors were increased and the levels of autophagy were decreased

**Figure 5:**
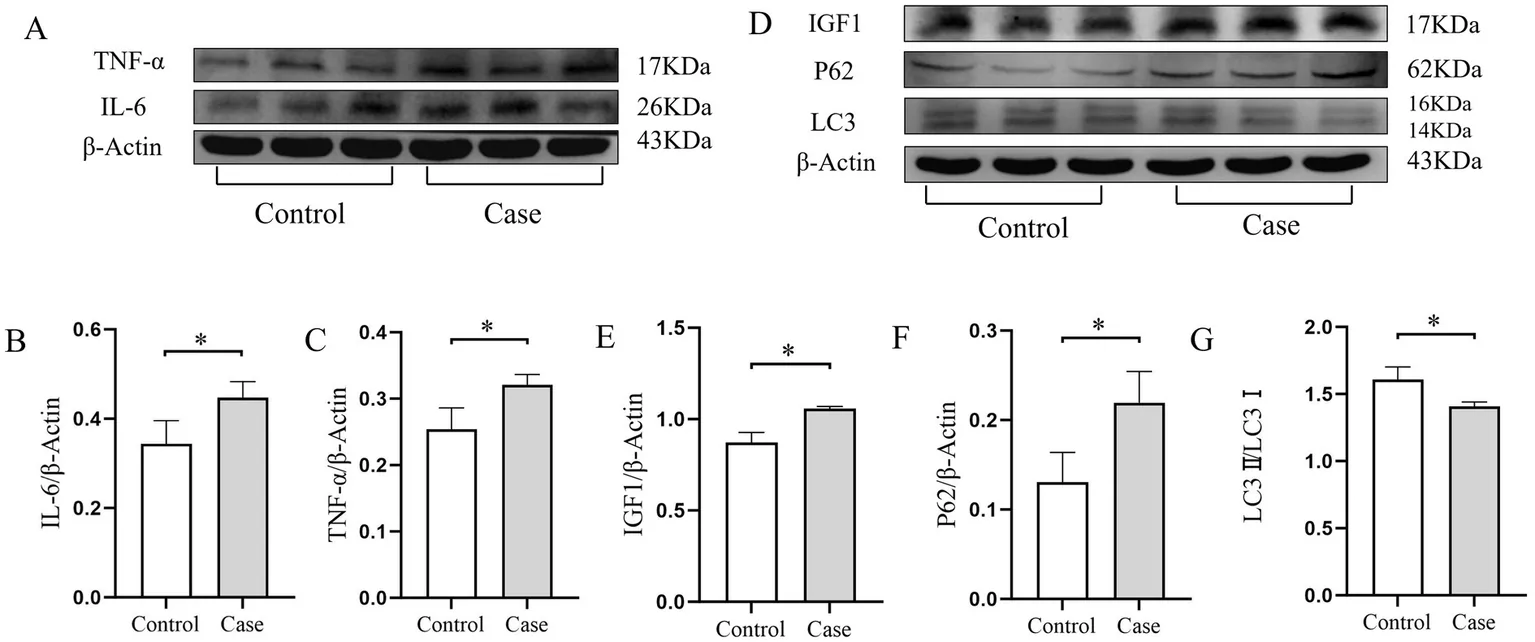
In the model group, the levels of inflammatory factors were increased and the levels of autophagy were decreased. **(A–C)** Western blot for TNF-α and IL-6 expression in the cell model stimulated by SiO_2_, β-actin was used as loading control. **(D–F)** Western blot for IGF1, P62, and LC3 expression in the cell model stimulated by SiO_2_, β-actin was used as loading control. Data are expressed as mean ± *SD* from at least three independent experiments in triplicate.\rCase: THP-1 cells were induced into macrophages by PMA and stimulated with SiO_2_ at 50 μg/mL. ^*^p < 0.05; ^**^p < 0.01; ^***^p < 0.001 versus the control group.

THP-1 cells were assigned to the control group and SiO₂-stimulated model group. The expression of inflammatory cytokines IL-6 and TNF-*α* was first measured, and the findings were consistent with the aforementioned ELISA data. Subsequent detection of IGF1 and autophagy-related proteins was further conducted. Compared with the control group, SiO₂-stimulated macrophages displayed elevated IGF1 levels, increased P62 accumulation, and a lower LC3II/LC3I ratio, with all intergroup differences statistically significant (*p* < 0.05) (Figure 5).

### 3.7 Elevated phosphorylation of PI3K and AKT in the model group

**Figure 6:**
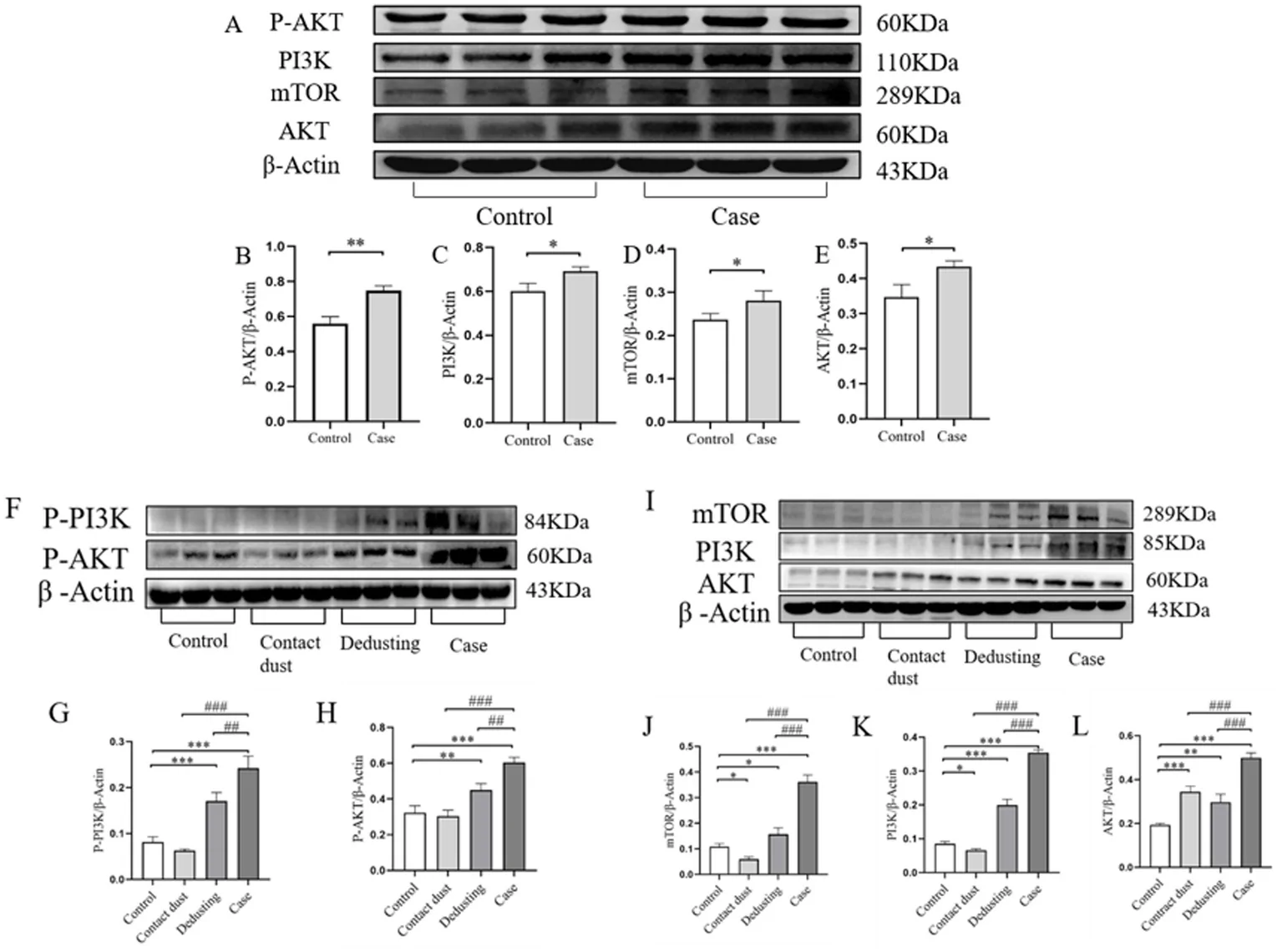
The PI3K/AKT/mTOR pathway was activated in the model group. **(A–E)** Western blot for P-AKT, PI3K, mTOR and AKT expression in the cell model stimulated by SiO_2_, β-actin was used as loading control. Data are expressed as mean ± SD from at least three independent experiments in triplicate. Case: THP-1 cells were induced into macrophages by PMA and stimulated with SiO_2_ at 50 μg/mL. **(F–H)** Western blot for P-AKT and P-PI3K expression in the human sample, β-actin was used as loading control. **(I–L)** Western blot for mTOR, PI3K and AKT expression in the human sample, β-actin was used as loading control. *p < 0.05; **p < 0.01; ***p < 0.001 versus the control group; #*p* < 0.05; ##*p* < 0.01; ###*p* < 0.001 versus the Case group.

Western blot was performed to detect the expression of PI3K/AKT/mTOR pathway-related proteins in THP-1 cells. Higher levels of phosphorylated PI3K (p-PI3K) and phosphorylated AKT (p-AKT) were observed in the SiO₂-stimulated model group relative to controls, with statistically significant intergroup differences (*p* < 0.05). These cellular results demonstrated elevated phosphorylation of PI3K and AKT, which showed synchronous expression changes with autophagy marker proteins. We further quantified PI3K, p-PI3K, AKT, p-AKT and downstream mTOR protein expression in peripheral blood samples from the mining population; consistent with cell-based findings, all these proteins were markedly elevated in the pneumoconiosis case group compared with the other three subgroups, reflecting consistent phosphorylation changes in human subjects. The matching protein expression trends between miner peripheral blood samples and SiO₂-challenged THP-1 cells suggest concurrent upregulation of IGF1, increased phosphorylation of PI3K and AKT, and altered autophagy-related protein expression in pneumoconiosis-related pathological changes (Figure 6).

## 4 Discussion

As reported in previous epidemiological research, progressive pulmonary fibrosis induced by long-term dust exposure renders pneumoconiosis a persistent global public health hazard (15). Current therapeutic regimens merely retard fibrotic progression via anti-fibrotic agents rather than reversing established lesions (14, 16, 17), and disease prevalence rises alongside aggravated fibrotic impairment (18). This intractable occupational disorder imposes severe threats to occupational population health and substantial socioeconomic burdens (19, 20). Against this clinical backdrop, identifying reliable serum biomarkers for early intervention and disease management carries prominent translational significance.

This study adopted a two-stage research design. First, we analyzed clinical data and biospecimens collected from coal mine workers to identify relevant risk factors for CWP, and characterized the expression changes of IGF1, inflammatory cytokines and autophagy markers across different dust-exposure subgroups. Second, we established an SiO₂-stimulated THP-1 macrophage model for *in vitro* validation. Western blot was used to detect PI3K/AKT/mTOR pathway-related proteins in both peripheral blood protein samples from miners and SiO₂-treated THP-1 macrophages. Combined population and cellular data revealed synchronous alterations in IGF1 expression, PI3K/AKT/mTOR phosphorylation activation, and the abundance of autophagy-related proteins in pneumoconiosis patients and silica-stimulated macrophages, indicating correlative molecular links among these molecules during CWP progression.

A variety of occupational, laboratory and lifestyle indicators are closely associated with coal workers’ pneumoconiosis and were treated as confounding factors in our analysis. Key occupational metrics including initial working age, cumulative dust exposure duration and total service years are well-recognized correlated factors for CWP (21); longer dust exposure periods increase pneumoconiosis risk, whereas circulating IGF1 levels tend to decrease with advancing worker age (22). The AST/ALT ratio, a liver function marker, was also considered because IGF1 is mainly secreted by the liver and this index may interfere with IGF1 measurements (23, 24). Peripheral WBC count reflects systemic inflammation, and robust inflammatory activation has been observed in pneumoconiosis patients (25), yet multiple physiological factors can alter inflammatory readouts and bias molecular experimental results. Smoking, an important lifestyle confounder, accelerates CWP progression and exacerbates respiratory disease prevalence when combined with dust exposure (26), while prior research confirms smoking alters circulating IGF1 concentrations and inflammatory responses (27–29). Collectively, smoking status, WBC levels, occupational length and liver function indicators may confound the relationships among dust exposure, IGF1 expression and pneumoconiosis, demonstrating that comprehensive confounding adjustment is essential for revealing the true association between dust-triggered IGF1 alterations and pneumoconiosis progression.

As a multifunctional regulatory molecule linked to multiple disease mechanisms (8, 30–32), IGF1 is closely involved in the dysregulated autophagy process that drives silica-induced pulmonary fibrosis in pneumoconiosis (33–35). It can be seen from bioinformatics analysis and protein interaction (36), IGF1 is highly expressed in pneumoconiosis. In addition, it has been reported that reduced IGF1 expression levels have been found in both *in vivo* and *in vitro* models of ischemic stroke (37), as an agonist of PI3K upstream of the pathway, IGF1 can activate the PI3K/AKT/mTOR pathway (37), thus inhibiting autophagy. Consistent with bioinformatics transcriptional prediction, human plasma and cellular experiments verified elevated IGF1 expression, together with altered autophagy-related protein expression, the upregulated transcription of PI3K/AKT/mTOR core genes predicted by transcriptome analysis combined with cellular protein detection results suggested that excessive activation of PI3K/AKT/mTOR signaling may form a potential linkage with inhibited autophagy in pneumoconiosis lesions (8, 9, 33, 37).

Integrating all the above analytical results, we elaborate the potential pathological mechanisms of pneumoconiosis based on population data and cellular evidence, respectively. At the population level, occupational and lifestyle risk factors including early entry into dust posts, long-term dust exposure and smoking do not only correlate with higher pneumoconiosis risk statistically, but may synergistically boost circulating IGF1 expression together with elevated WBC and abnormal AST/ALT ratio; along the disease progression from dust-exposed populations to confirmed pneumoconiosis patients, elevated IGF1 was accompanied by activated PI3K/AKT/mTOR cascade, changes in autophagy-related proteins and persistent systemic inflammation, forming a progressive profibrotic loop that may explain the continuous deterioration of lung lesions even after dust withdrawal, while liver function status alters basal IGF1 secretion and may further amplify this pathological process. At the cellular level, SiO₂-stimulated THP-1 macrophage models excluded population confounding factors and reproduced the same synchronous molecular expression trends observed in human peripheral blood samples. We observed correlative sequential changes: silica exposure was accompanied by IGF1 upregulation, concurrent elevated PI3K/AKT phosphorylation and increased mTOR protein levels, together with altered LC3 and accumulated P62, and residual dust and necrotic cell fragments could continuously induce IL-6 and TNF-*α* release to form a self-perpetuating inflammatory-fibrotic cycle. Collectively, this work characterizes the correlative IGF1-PI3K/AKT/mTOR-autophagy molecular axis linked to silica-triggered lung injury and illustrates how this molecular cascade participates in the development of coal workers’ pneumoconiosis.

However, this study also has several limitations. First, this is a cross-sectional investigation, and occupational and lifestyle information relied on self-reporting, which may introduce recall bias; moderate multicollinearity also existed among age, initial working age, dust exposure duration and total seniority, restricting independent interpretation of each time-related covariate despite VIF < 10. Second, our findings are correlative, as we did not perform IGF1 manipulation or pathway intervention experiments. Additionally, the lack of paired phosphorylated and total protein measurements from the same batches precluded calculation of p-PI3K/PI3K and p-AKT/AKT ratios. Therefore, our data indicate elevated phosphorylation levels of PI3K and AKT that correlate with changes in autophagy-related proteins, rather than definitive pathway activation. Third, all subjects were recruited from a single coal mine, which may limit the generalizability of our conclusions, and large prospective cohorts are needed for external verification.

Despite the above deficiencies, this study systematically combined population epidemiological data and *in vitro* cell detection to characterize the alterations of IGF1, autophagy and downstream signaling molecules under silica exposure. Our results provide new correlative evidence between IGF1, PI3K/AKT/mTOR signaling and coal workers’ pneumoconiosis, and offer potential serum indicators for pneumoconiosis population screening, laying a preliminary theoretical basis for subsequent research on intervention targets against pneumoconiosis fibrosis.

## Data Availability

The original contributions presented in the study are included in the article/supplementary material, further inquiries can be directed to the corresponding author/s.

## References

[1] Perret JL Plush B Lachapelle P Hinks TSC Walter C Clarke P . Coal mine dust lung disease in the modern era. Respirology. (2017) 22:662–70. doi: 10.1111/resp.13034, 28370783

[2] Almberg KS Friedman LS Rose CS Go LHT Cohen RA. Progression of coal workers' pneumoconiosis absent further exposure. Occup Environ Med. (2020) 77:748–51. doi: 10.1136/oemed-2020-106466, 32788293

[3] Go LHT Cohen RA. Coal workers' pneumoconiosis and other mining-related lung disease: new manifestations of illness in an age-old occupation. Clin Chest Med. (2020) 41:687–96. doi: 10.1016/j.ccm.2020.08.002, 33153687

[4] Ogawa T Shichino S Ueha S Bando K Matsushima K. Profibrotic properties of C1q(+) interstitial macrophages in silica-induced pulmonary fibrosis in mice. Biochem Biophys Res Commun. (2022) 599:113–9. doi: 10.1016/j.bbrc.2022.02.037, 35180470

[5] Liu H He H Tian Y Cui J Wang S Wang HL. Cyclophilin a accelerates SiO(2)-induced macrophage foaming. Cell Signal. (2023) 103:110562. doi: 10.1016/j.cellsig.2022.110562 36535629

[6] Wang XC Song K Tu B Sun H Zhou Y Xu SS . New aspects of the epigenetic regulation of Emt related to pulmonary fibrosis. Eur J Pharmacol. (2023) 956:175959. doi: 10.1016/j.ejphar.2023.175959, 37541361

[7] Du S Li C Lu Y Lei X Zhang Y Li S . Dioscin alleviates crystalline silica-induced pulmonary inflammation and fibrosis through promoting alveolar macrophage autophagy. Theranostics. (2019) 9:1878–92. doi: 10.7150/thno.29682, 31037145 PMC6485284

[8] Werner H. The Igf1 signaling pathway: from basic concepts to therapeutic opportunities. Int J Mol Sci. (2023) 24:14882. doi: 10.3390/ijms241914882, 37834331 PMC10573540

[9] Wang H Liu Y Wang D Xu Y Dong R Yang Y . The upstream pathway of mtor-mediated autophagy in liver diseases. Cells. (2019) 8:1597. doi: 10.3390/cells8121597, 31835352 PMC6953127

[10] The National Essential Public Health Service Program Office for Management of Hypertension in Primary Health Care, National Center for Cardiovascular Diseases, National Committee on Hypertension Management in Primary Health Care. National clinical practice guidelines on the management of hypertension in primary health care in China (2020). Chin J Front Med Sci. (2021) 13:26–37.

[11] Sun Q Ren Q Du L Chen S Wu S Zhang B . Cardiometabolic index (cmi), lipid accumulation products (lap), waist triglyceride index (Wti) and the risk of acute pancreatitis: a prospective study in adults of North China. Lipids Health Dis. (2023) 22:190. doi: 10.1186/s12944-023-01948-3, 37946249 PMC10633920

[12] Li X Xu X Song Y Cui S Xue C Wang L . An association between cumulative exposure to light at night and the prevalence of hyperuricemia in steel workers. Int J Occup Med Environ Health. (2021) 34:385–401. doi: 10.13075/ijomeh.1896.01648, 33559651

[13] Li X Cui S Wu J Wang L Yuan J. Job category differences in the prevalence and associated factors of insomnia in steel workers in China. Int J Occup Med Environ Health. (2020) 33:215–33. doi: 10.13075/ijomeh.1896.01519, 32162617

[14] Wang NN Jin Y Wang CX. Research advances in imaging techniques related to the diagnosis of pneumoconiosis. Zhonghua Lao Dong Wei Sheng Zhi Ye Bing Za Zhi. (2023) 41:876–80. doi: 10.3760/cma.j.cn121094-20220304-00110, 37935559

[15] Wang D Liang R Yang M Ma J Li W Mu M . Incidence and disease burden of coal workers' pneumoconiosis worldwide, 1990-2019: evidence from the global burden of disease study 2019. Eur Respir J. (2021) 58:2101669. doi: 10.1183/13993003.01669-2021, 34561283

[16] Weissman DN. Progressive massive fibrosis: an overview of the recent literature. Pharmacol Ther. (2022) 240:108232. doi: 10.1016/j.pharmthera.2022.108232, 35732247 PMC10053429

[17] Lurje I Gaisa NT Weiskirchen R Tacke F. Mechanisms of organ fibrosis: emerging concepts and implications for novel treatment strategies. Mol Asp Med. (2023) 92:101191. doi: 10.1016/j.mam.2023.101191, 37236017

[18] Deng LH Wang ZP. The prevalence of common complications among pneumoconiosis patients: a systematic review and Meta-analysis. Zhonghua Lao Dong Wei Sheng Zhi Ye Bing Za Zhi. (2023) 41:931–8. doi: 10.3760/cma.j.cn121094-20230118-00018, 38195231

[19] Qi XM Luo Y Song MY Liu Y Shu T Liu Y . Pneumoconiosis: current status and future prospects. Chin Med J. (2021) 134:898–907. doi: 10.1097/CM9.0000000000001461, 33879753 PMC8078400

[20] Hu W Wu WN Qiao Q. Occupational survey-based evidence of health status and welfare problems of workers with pneumoconiosis in China. Front Public Health. (2023) 11:1142161. doi: 10.3389/fpubh.2023.1142161, 37719739 PMC10501603

[21] Naidoo RN Jeebhay MF. Covid-19: a new burden of respiratory disease among south African miners? Curr Opin Pulm Med. (2021) 27:79–87. doi: 10.1097/MCP.0000000000000759, 33417344 PMC7924928

[22] Iwasaki K Lalani B Kahng J Carapeto P Sanjines S Hela F . Decreased Igf1R attenuates senescence and improves function in pancreatic β-cells. Front Endocrinol (Lausanne). (2023) 14:1203534. doi: 10.3389/fendo.2023.1203534, 37441495 PMC10335398

[23] Kineman RD Del Rio-Moreno M Waxman DJ. Liver-specific actions of Gh and Igf1 that protect against Masld. Nat Rev Endocrinol. (2024) 21:105–17. doi: 10.1038/s41574-024-01037-0, 39322791

[24] Chinnappan R Mir TA Alsalameh S Makhzoum T Alzhrani A Al-Kattan K . Low-cost point-of-care monitoring of alt and Ast is promising for faster decision making and diagnosis of acute liver injury. Diagnostics. (2023) 13. doi: 10.3390/diagnostics13182967, 37761334 PMC10529728

[25] Yoon EC Koo SM Park HY Kim HC Kim WJ Kim KU . Predictive role of white blood cell differential count for the development of acute exacerbation in Korean chronic obstructive pulmonary disease. Int J Chron Obstruct Pulmon Dis. (2024) 19:17–31. doi: 10.2147/COPD.S435921, 38192972 PMC10773455

[26] Colarusso C Falanga A Di Caprio S . The activation of the Aim2 inflammasome after cigarette smoke exposure leads to an immunosuppressive lung microenvironment. Int Immunopharmacol. (2024) 131:111832. doi: 10.1016/j.intimp.2024.111832, 38460301

[27] Yıldırım BB Kulaksızoglu S. Prolidase could be considered a sign of inflammation associated with cigarette smoking. Front Med. (2024) 11:1347688. doi: 10.3389/fmed.2024.1347688, 38638929 PMC11024229

[28] Wang F Li H Kong T Shan L Guo J Wu Y . Association of cigarette smoking with cerebrospinal fluid biomarkers of insulin sensitivity and neurodegeneration. Brain Behav. (2024) 14:e3432. doi: 10.1002/brb3.3432, 38361318 PMC10869886

[29] Koh DH Choi S Park JH Lee SG Kim HC Kim I . Evaluation on the sex-specific association between cigarette smoke exposure and inflammation markers-C-reactive protein and white blood cell count. Nicotine Tob Res. (2024) 26:484–93. doi: 10.1093/ntr/ntad182, 37742212

[30] Park K Lee MS. Current status of autophagy enhancers in metabolic disorders and other diseases. Front Cell Dev Biol. (2022) 10:811701. doi: 10.3389/fcell.2022.811701, 35237600 PMC8882819

[31] Liu L Li X. A review of Igf1 signaling and Igf1-related long noncoding Rnas in chemoresistance of cancer. Curr Cancer Drug Targets. (2020) 20:325–34. doi: 10.2174/1568009620666200228123754, 32186268

[32] Wang J Zhu Q Cao D Peng Q Zhang X Li C . Bone marrow-derived Igf-1 orchestrates maintenance and regeneration of the adult skeleton. Proc Natl Acad Sci USA. (2023) 120:e2203779120. doi: 10.1073/pnas.2203779120, 36577075 PMC9910602

[33] Zhao H Wang Y Qiu T Liu W Yao P. Autophagy, an important therapeutic target for pulmonary fibrosis diseases. Clin Chim Acta. (2020) 502:139–47. doi: 10.1016/j.cca.2019.12.016, 31877297

[34] Park W Wei S Kim BS Kim B Bae SJ Chae YC . Diversity and complexity of cell death: a historical review. Exp Mol Med. (2023) 55:1573–94. doi: 10.1038/s12276-023-01078-x, 37612413 PMC10474147

[35] Li W Xie L Ma J Cheng M Fan L Xu Y . Gas6 or Mer deficiency ameliorates silica-induced autophagosomes accumulation in mice lung. Toxicol Lett. (2021) 337:28–37. doi: 10.1016/j.toxlet.2020.11.013, 33232774

[36] Szklarczyk D Kirsch R Koutrouli M Nastou K Mehryary F Hachilif R . The String database in 2023: protein-protein association networks and functional enrichment analyses for any sequenced genome of interest. Nucleic Acids Res. (2023) 51:D638–46. doi: 10.1093/nar/gkac1000, 36370105 PMC9825434

[37] Yoshida T Delafontaine P. Mechanisms of Igf-1-mediated regulation of skeletal muscle hypertrophy and atrophy. Cells. (2020) 9:1970. doi: 10.3390/cells9091970, 32858949 PMC7564605

